# Functional Characterization of Glucokinase Variants to Aid Clinical Interpretation of Monogenic Diabetes

**DOI:** 10.3390/ijms27010156

**Published:** 2025-12-23

**Authors:** Varsha Rajesh, Dora Evelyn Ibarra, Jing Yang, Haichen Zhang, Amy Barrett, Eleanor G. Kaplan, Amit Kumthekar, Fanny Sunden, Han Sun, Ananta Addala, Aaron Misakian, Lisa R. Letourneau-Freiberg, Colleen O. Jodarski, Kristin A. Maloney, Cécile Saint-Martin, Polly M. Fordyce, Toni I. Pollin, Anna L. Gloyn

**Affiliations:** 1 Department of Pediatrics, Division of Endocrinology, Stanford School of Medicine, Stanford, CA 94305, USA; vrajesh@stanford.edu (V.R.); evelyn.ibarra@cuanschutz.edu (D.E.I.); jingy6@stanford.edu (J.Y.); egkaplan@stanford.edu (E.G.K.); amitsk@stanford.edu (A.K.); hansun@stanford.edu (H.S.); aaddala@stanford.edu (A.A.); or kmg7uf@uvahealth.org (A.M.); 2 Barbara Davis Center for Diabetes, University of Colorado Anschutz Medical Campus, Aurora, CO 80045, USA; 3 Department of Medicine, University of Maryland School of Medicine, Baltimore, MD 21201, USA; zhanghaichen@genomics.cn (H.Z.); colleen.jodarski@som.umaryland.edu (C.O.J.); kmaloney1@som.umaryland.edu (K.A.M.); tpollin@som.umaryland.edu (T.I.P.); 4 Oxford Centre for Diabetes, Endocrinology and Metabolism, University of Oxford, Churchill Hospital, Oxford OX3 7LE, UK; amy.barrett@drl.ox.ac.uk; 5 Department of Genetics, Stanford School of Medicine, Stanford, CA 94305, USA; pfordyce@stanford.edu; 6 Department of Biochemistry, Stanford School of Medicine, Stanford, CA 94305, USA; fsunden@stanford.edu; 7 Kovler Diabetes Center, University of Chicago, Chicago, IL 60637, USA; lletourneaufreiberg@bsd.uchicago.edu; 8 Department of Medical Genetics, AP-HP Pitié-Salpêtrière Hospital, Sorbonne University, 75012 Paris, France; cecile.saint-martin@aphp.fr; 9 Department of Bioengineering, Stanford University, Stanford, CA 94305, USA; 10 Sarafan ChEM-H, Stanford, CA 94305, USA; 11 Chan Zuckerberg Biohub, San Francisco, CA 94158, USA; 12 Stanford Diabetes Research Center, Stanford School of Medicine, Stanford, CA 94305, USA

**Keywords:** glucokinase, MODY, monogenic diabetes, VUS, hypoglycemia

## Abstract

Precision medicine starts with a precision diagnosis. Yet up to 80% of cases of monogenic diabetes, a form of diabetes characterized by mutations in a single gene, are either overlooked or misdiagnosed. A genetic test for monogenic diabetes does not always lead to a precise diagnosis, as novel variants are often classified as variants of unknown significance. Variant interpretation requires collation of a framework of evidence, including population, computational, and segregation data, and can be assisted by functional analysis. The inclusion of functional data can be challenging, depending on the number of benign and pathogenic variants available for benchmarking assays. Glucokinase is the rate-limiting step for glucose metabolism in the pancreatic beta-cell and governs the threshold for glucose-stimulated insulin release. Loss-of-function alleles in the glucokinase (*GCK*) gene are a cause of stable fasting hyperglycemia from birth and/or diabetes. In this study, we functionally characterized 25 variants identified during diagnostic testing or in exome sequencing studies. We assessed their kinetic characteristics, stability, and interaction with pharmacological and physiological regulators. We integrated our functional data with existing data from the ClinGen Monogenic Diabetes Variant Curation Expert Review panel using a gene-specific framework to assist variant classification. We show how functional evidence can aid variant classification, thus enabling diagnostic certainty.

## 1. Introduction

Monogenic diabetes is a rare subtype of diabetes caused by defects in a single gene, and accounts for up to 5% of all diabetes cases diagnosed under the age of 40 [1,2]. It encompasses a range of conditions, including maturity-onset diabetes of the young (MODY), permanent and transient neonatal diabetes (PNDM and TNDM, respectively), and various syndromic forms [3]. Over 40 different genetic etiologies have been described, and there is established evidence that a genetic diagnosis informs optimal treatment, prognosis, and risk for family members [3,4]. Individuals with monogenic forms of diabetes are often misdiagnosed as having either type 1 or type 2 diabetes, for which insulin and metformin, respectively, are often the first line of treatment, leading to sub-optimal management of their diabetes and missed opportunities to counsel family members. Access to genetic testing varies greatly between and within countries, and even when testing is available, there are challenges with the interpretation of rare variants, especially in understudied populations [4].

One of the most common genes implicated in monogenic diabetes encodes the enzyme glucokinase, which is a member of the hexokinase family of enzymes [5,6,7]. Glucokinase has unique kinetic characteristics which enable it to govern the threshold for glucose-stimulated insulin secretion (GSIS) in pancreatic beta cells and glucose storage in the liver [8]. Genetic variation in the glucokinase gene (*GCK*) can change the threshold for GSIS, resulting in either elevated fasting glucose levels and/or diabetes or inappropriate secretion of insulin at low blood glucose levels [7,9,10]. Heterozygous inactivating (loss of function; LoF) alleles in *GCK* cause elevated fasting plasma glucose levels or GCK-MODY, while activating (gain of function; GoF) alleles cause the opposite phenotype of persistent hyper-insulinemic hypoglycemia of infancy (PHHI) [7,9]. Homozygous and compound heterozygous inactivating variants have also been reported, and depending on their functional severity, can cause either PNDM or GCK-MODY [7,10,11]. The phenotype of individuals with heterozygous LoF *GCK* variants is remarkably similar, even when the functional severity of the variant is very different [12]. This can be explained by a glucose-induced post-translational upregulation of the wild-type allele, which compensates for the dysfunctional allele [13,14].

In 2015, the American College of Medical Genetics and Genomics (ACMG) and Association for Molecular Pathology (AMP) updated guidelines on interpreting sequence variants in Mendelian disorders to include specific standard terminology of five classifications (from “benign” to “pathogenic”) and a classification pipeline using a framework of evidence with various weights, including functional data along with clinical case data, population database frequency, familial co-segregation data, in silico predictive tools, information about functional domains/mutational hotspots, and knowledge about other variants affecting the same amino acid or nucleotide [15]. The NIH-funded Clinical Genome Resource (ClinGen) has expanded and updated these guidelines. For example, Brnich and colleagues published a framework for evaluating evidence from in vitro studies [12], while the original ACMG/AMP guidelines proposed a default strong weight for functional evidence using the criterion codes PS3/BS3, Brnich et al. defined a rigor for functional studies as either having a specified number of positive and negative controls or validating proposed cutoffs for functional assays for their ability to separate pathogenic and benign variants using an odds path metric [16].

ClinGen also established oversight of numerous disease- and gene-specific Variant Curation Expert Panels (VCEPs) [17]. The ClinGen Monogenic Diabetes Variant Curation Expert Panel (MDEP) was established in 2017 and has developed and published gene-specific rules for *HNF1A*, *HNF4A*, *GCK*, and monogenic diabetes, with others in progress [18]. The component of the *GCK*-specific rules was developed using the Brnich framework [16]. Despite the generation of deep mutational scanning datasets for *GCK*, there remain challenges with interpreting missense variants, demonstrating the need for additional approaches to fully capture the suite of molecular mechanisms for enzyme dysfunction [19]. Gold standard in vitro kinetic assays, which assess GCK’s activity, stability, and interaction with physiological and pharmacological regulators, provide a comprehensive assessment of enzyme function and have been modeled to predict the in vivo threshold for GSIS [14,20]. For a small number of variants, the assays have been benchmarked against cellular (pancreatic beta-cell) assays, demonstrating concordance [21,22]. Critically, they can discern between variants with complex molecular mechanisms [11,23,24] and those that are benign [25,26]. Surprisingly few benign variants have been established, and most variants that have been comprehensively characterized have been shown to affect enzyme function, making it challenging to implement the Brnich framework [16,25,26].

**Figure 1:**
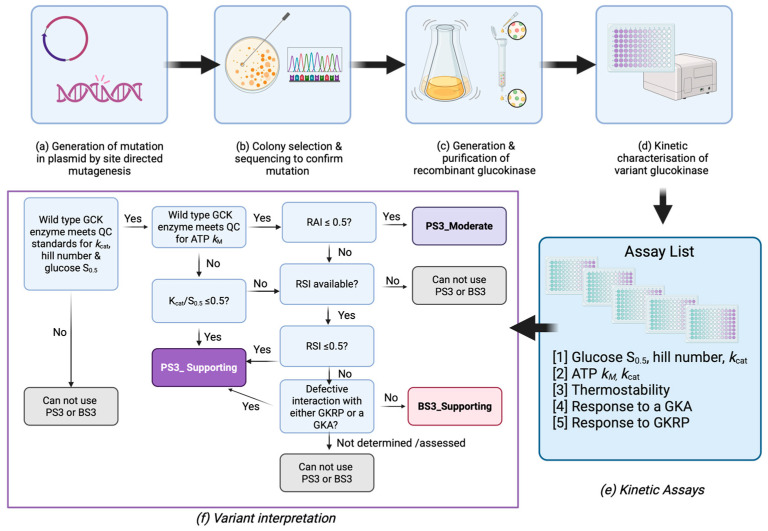
Schematic representation of the pipeline for the interpretation of functional evidence for GCK variants. (**a**) Variants are generated by site-directed mutagenesis of a GST-tagged human islet *GCK* cDNA. (**b**) Mutations are confirmed by Sanger sequencing. (**c**) Recombinant human GCK protein is made in *E. coli* and purified by its GST-tag. (**d**) Recombinant enzymes are kinetically characterized by measuring enzymatic activity at different substrate concentrations. (**e**) Up to five different assays are performed to characterize the enzyme behavior, stability, and interactions with physiological (glucokinase regulatory protein; GKRP) or pharmacological (glucokinase activator; GKA) regulators. (**f**) MDEP VCEP decision tree for classification of variants based on functional evidence. Glucose S_0.5_; affinity for substrate glucose, Hill number; cooperativity of GCK with respect to its substrate glucose, *k_cat_*; maximal specific activity, ATP *k*_M_; affinity for substrate ATP, RAI; relative activity index of variant with respect to wild-type, RSI; relative stability index with respect to wild-type, PS3_Moderate; Moderate level criterion based on functional evidence in favor of pathogenicity, PS3_Supporting; Supporting level criterion based on functional evidence in favor of pathogenicity, BS3_Supporting; Supporting level criterion based on functional evidence in favor of benignity.

We worked with our MDEP collaborators to identify cutoffs for in vitro kinetic and thermostability data using published data by us and others that enabled the inclusion of functional evidence for variant classification. MDEP used the Brnich framework to evaluate an observation-derived evidence and applied cutoffs of 0.5 for pathogenicity to assess the ability of the RAI to distinguish between variants identified as pathogenic/likely pathogenic (n = 17) or benign/likely benign (n = 5) before application of in vitro data and were able to derive an odds of pathogenicity of 7.94 for variants with RAI < 0.5. Application of this evaluation required that wild-type data fall within the QC ranges (*k*_cat_ = 40–80, S_0.5_ = 6.0–9.0, Hill number = 1.4–1.8, and ATP *k*_M_ = 0.2–0.5). For assays with wild-type ATP *k*_M_ outside the QC range, they constructed an ROC curve based on existing data for the relative *k_cat_*/S_0.5_ ratio and found that a value of 0.5 maximized sensitivity and specificity to allow the application of PS3_Supporting. Variants with RAI > 0.5 or relative *k_cat_*/S_0.5_ ratio were further evaluated using RSI and GKRP/GKA interactions as specified in Figure 1. The limited number of data points enabled the application of PS3_Supporting for those variants with RSI ≤ 0.5 or observed impact on GKRP/GKA interactions and BS3_Supporting for those variants with RAI > 0.5, RSI > 0.5, and no impact on GKRP/GKA interaction.

The aim of this study was to perform comprehensive functional studies on missense variants identified in a published exome sequencing project, which included subjects with and without type 2 diabetes, and variants of unknown significance (VUS) reported by diagnostic labs to assist their interpretation in diagnostic testing.

## 2. Results

### 2.1. Functional Characterization of GCK Variants Using Gold Standard In Vitro Assays

Details of the 25 GCK variants evaluated in this study are described in Table 1 and Table 2. Twenty of them (all missense) were identified via an exome sequencing study of 12,940 individuals from the T2D-GENES study [27]. The remaining five were identified during routine diagnostic testing for monogenic diabetes and referred to the MDEP for interpretation. Twenty-three variants are missense, one is an insertion, and one is a deletion. All variants were functionally characterized using gold-standard substrate titration assays to measure the variant enzyme’s affinity for glucose and ATP, the cooperativity constant, as well as enzyme velocity (Figure 1). Out of 25 variants, 11 showed reduced activity compared to that of wild-type GCK—either through an increased glucose S_0.5_, increased ATP *k*_M_, or decreased *k*_cat_. Out of the 11, 3 were completely inactive and did not respond to any concentration of substrate. These 11 variants had a relative activity index (RAI) lower than 0.5, and according to the decision tree (Figure 1f), the criterion of PS3_Moderate could be granted.

**Figure 2:**
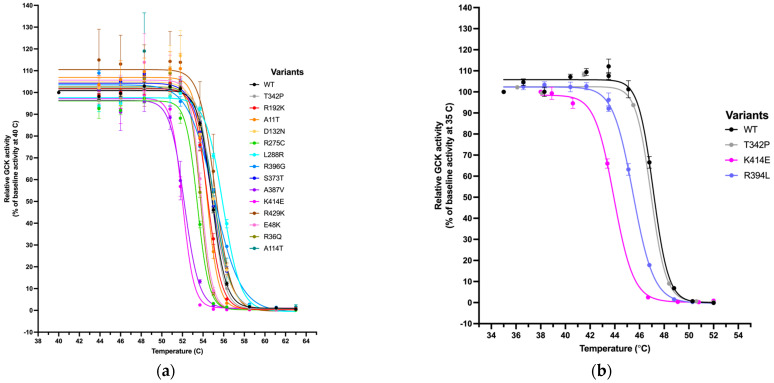
Assessment of thermostability for GCK-variants. Relative activity normalized to activity at baseline temperature for WT-GCK and 14 GCK variants, as a function of increasing temperature. Thermolability assays were performed in the presence of 8 mM glucose and saturating amounts of ATP (mM). T342P was used as a WT-like control, while K414E was run in all assays to establish lab-to-lab reproducibility. Curves were fit to a four-parameter logistic regression model in GraphPad PRISM 10. (**a**) Variants assessed in Oxford (from the T2DGENES study). Activity was assessed at a temperature range of 40 to 63 °C. (**b**) Variants assessed in Stanford (from clinical diagnostic labs). Activity was assessed at a temperature range of 35 to 52 °C. Residual plots can be found in Supplementary Figure S1.

The remaining 14 variants required further characterization and were assessed for their stability compared to that of wild-type (WT) (described by TA50, Table 1). It is known that *GCK* variants can mediate their effects on enzyme function through altering stability, as some pathogenic *GCK* variants exhibit either normal or paradoxical in vitro kinetic profiles [11,22,23,24,28]. Effects of temperature on enzyme activity (as a proxy for protein stability) were evaluated, and the TA50 (the temperature at which GCK performs at half its baseline temperature activity) was calculated (Figure 2a,b). A low TA50 indicates that the variant is more thermostable. The relative stability index (RSI) for the remaining variants was assessed by normalizing the TA50 of the variants to that of wild-type and the least stable variant, K414E. Three variants had an RSI lower than 0.5 and could be classified as PS3_Supporting according to the decision tree (Figure 1f). This indicated the variant exhibited pathogenic characteristics, but not as strongly as required for a PS3_Moderate classification, and that the pathogenicity was mediated primarily through effects on protein stability. A fourth variant, R275C, had an RSI of 0.5. Since it was on the cutoff threshold for this assay, we took it forward to assess its interaction with binding partners.

**Figure 3:**
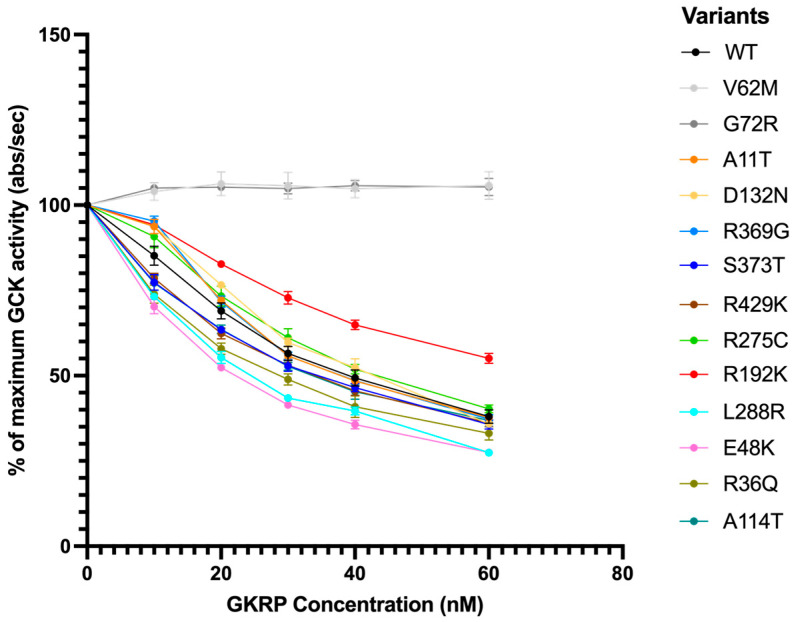
Effect of GKRP binding on the maximum activity of GCK-WT and 11 variant GCK proteins. Two variants (V62M and G72R) previously reported to have little to no interaction with GKRP are included as negative controls, shown in light and dark gray. Activity has been normalized to activity at 0 mM GKRP. The glucose and ATP concentrations in the assay are 3 mM and 5 mM, respectively. The average of 3 technical replicates is shown per variant, 10 for WT-GCK, and 6 and 9 for the V62M-GCK and G72R-GCK, respectively.

The remaining 11 variants showed either normal or increased stability compared to wild-type, based on their RSI, and required assessment of their interactions with a physiological inhibitor (glucokinase regulatory protein; GKRP) and a pharmacological activator (GKA) to make a classification. GKRP is primarily found in hepatocytes and regulates the translocation of GCK across the nucleus as well as its ability to bind glucose [29]. GKRP competes with glucose to bind in the glucose-binding pocket; therefore, at high concentrations of glucose, GKRP is outcompeted, resulting in increased GCK activity and glucose metabolism. At low concentrations of glucose, GKRP can bind to and inhibit GCK, allowing for homeostasis to shift in the direction of glycogen breakdown and glucose release into the bloodstream. Any effect on GKRP binding would have implications for the variant’s ability to contribute to glucose metabolism and homeostasis in the liver. Our results for all 11 variants are shown in Figure 3. WT-GCK activity decreases by about 50% with the addition of 60 nM GKRP, while the controls V62M-GCK and G72R-GCK are unaffected by GKRP as expected [23,24]. All other variants are inhibited similarly by GKRP, indicating that the capacity to bind GKRP is preserved in these variant structures. R192K-GCK exhibited an upward shift in activity compared to WT-GCK, consistent with a mild decrease in GKRP affinity.

**Figure 4:**
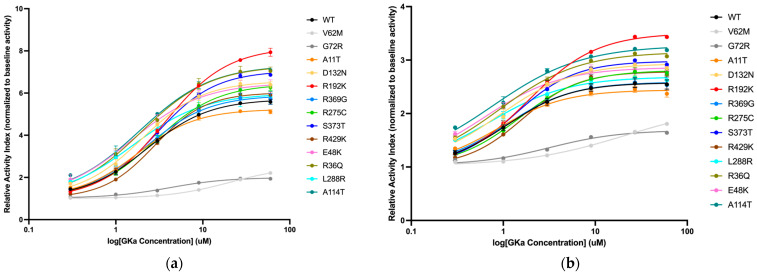
Effect of allosteric GKA RO-28-1675 on the activity index of glucokinase wild-type and 11 variants. V62M-GCK and G72R-GCK are included in light and dark gray as negative controls. Relative activity has been normalized to baseline activity at 0 mM GKA. Curves have been fit in Graphpad PRISM 10 using a Sigmoidal 4PL regression model, with GKA concentration transformed to log_10_. (**a**) Glucose concentration in the assay is 3.125 mM. The average of three technical replicates per variant is shown, except for A114T, which has n = 2. (**b**) Glucose concentration in the assay is 6.25 mM. The average of three technical replicates per variant is shown.

A novel class of small molecular activators for GCK (GKA) was first described in the early 2000s, with one of the first being RO-28-1675, the potent and active R enantiomer of RO-28-0450 [30]. RO-28-1675 is thought to bind to an allosteric activator site on GCK, ∼20 Å from the glucose-binding pocket [31]. We characterized the effect of RO-28-1675 on all 11 variants by titrating different concentrations of activators at different glucose concentrations (Figure 4). Two previously studied pathogenic GCK variants (V62M and G72R) were included as controls and had consistently higher EC50s in line with previously published data showing that they are not responsive to GCK activators [23,24]. The EC50 of the drug for WT-GCK is 2.30 ± 0.44 nM. Compared to WT-GCK, the remaining variants had similar EC50s and fold activations, indicating that the drug had a similar effect on the variants as it did on wild-type. All 11 variants assessed responded to the activator (Figure 4).

### 2.2. Classification of GCK Variants Using the Monogenic Diabetes VCEP Framework

*GCK* variant classifications made in line with the ACMG guidelines take into account genetic evidence, family history, population data, clinical testing, and in silico predictions. Descriptions of these classification criteria (such as PVS1, PS4, PM2, PP1, etc.) are detailed in the ClinGen Monogenic Diabetes Expert Panel (MDEP) Specifications to the ACMG/AMP Variant Interpretation Guidelines for GCK, Version 3.1.0 [18]. This Criteria Specification Registry also details rules for combining criteria to obtain a final classification (Pathogenic, Likely Pathogenic, Likely Benign, Benign, and finally VUS if not enough information is present). Although on their own, neither in vitro nor in vivo functional data are sufficient to classify a variant, they play an important role in assisting variant interpretation (coded as PS3 or BS3 in the Specifications) [15]. ClinVar has reported 19 of the 25 variants, but in most cases, these have not been aligned with ACMG guidelines and have conflicting classifications (Supplementary Table S1). We therefore focused on the variants reported in the ClinGen database, which have been classified by the MDEP VCEP panel. For the 25 variants in our study, 9 have previously been evaluated and are currently classified by ClinGen (Table 2). We deployed the MDEP VCEP GCK PS3/BS3 decision tree (Figure 1f) and assessed how the availability of functional data refined variant interpretation (Table 2). For 8 of the 10 variants previously classified by MDEP, the functional evidence does not alter their classification. For the remaining two, the addition of functional data allows reclassification from VUS to likely pathogenic. For the 16 variants that have not yet been evaluated by MDEP, our functional data gave 8 variants a BS3_Supporting criterion and 8 variants a PS3_Moderate criterion.

**Table 2:** Summary of variants evaluated in the study (GCK transcript NM_000162.5) and their clinical interpretation with and without the inclusion of functional data.

Variant (HGVS)	1-Letter Code	Genomic Position (GRCh38)	Molecular Consequence	MDEP/VCEP Classification Without PS3/BS3 Criteria If Available	Met Codes	PS3/BS3 Interpretation Using Functional Evidence (Figure 1f)	MDEP Classification with PS3/BS3 Criteria
c.31G>A (p.Ala11Thr)	A11T	chr7:44188923:C:T	missense	Benign	BA1	BS3_Supporting	Benign
c.107G>A (p.Arg36Gln)	R36Q	chr7:44153402:C:T	missense	Not reported	PP2,PP3	BS3_Supporting	-
c.142G>A (p.Glu48Lys)	E48K	chr7:44153367:C:T	missense	Likely benign	PP2, BS2, BP2	BS3_Supporting	Likely benign
c.340G>A (p.Ala114Thr)	A114T	chr7:44152294:C:T	missense	Not reported	PM2_S, PP2, PP3	BS3_Supporting	-
c.394G>A (p.Asp132Asn)	D132N	chr7:44151045:C:T	missense	Not reported	PP2	BS3_Supporting	-
c.469G>A (p.Glu157Lys)	E157K	chr7:44150970:C:T	missense	Not reported	PS4, PM2_S, PP2	PS3_Moderate	-
c.509_517dup (p.Gly170_Lys172dup) **	G170_K172dup	N/A	insertion	VUS	PP4_M PM2_S PM4	PS3_Moderate	Likely pathogenic
c.562G>A (p.Ala188Thr)	A188T	chr7:44149986:C:T	missense	Not reported	PP2, PP3	PS3_Moderate	-
c.575G>A (p.Arg192Lys)	R192K	chr7:44149973:C:T	missense	Not reported	PM2, PP3	BS3_Supporting	-
c.608T>C (p.Val203Ala)	V203A	chr7:44149831:A:G	missense	Pathogenic	PP4_M, PP1_S, PM2_S, PS3_M, PS4, PP2, PP3	PS3_Moderate	Pathogenic
c.638_640delGCT ** (p.Cys213del)	C213del	chr7:44149799:AGC:-	deletion	VUS	PP4_M, PM2_S, PM4_S	PS3_Moderate	Likely pathogenic
c.676G>A (p.Val226Met)	V226M	chr7:44149763:C:T	missense	Pathogenic	PP4_M, PP1_St, PM2_S, PS3_M, PS4, PM1, PP2, PP3	PS3_Moderate	Pathogenic
c.716A>G (p.Gln239Arg)	Q239R	chr7:44147797:T:C	missense	Not reported	BA1, PP2	PS3_Moderate	-
c.772G>A (p.Gly258Ser)	G258S	chr7:44147741:C:T	missense	Not reported	PP2, PP3, PM1, PM2_S	PS3_Moderate	-
c.773G>A (p.Gly258Asp)	G258D	chr7:44147740:C:T	missense	Not reported	PP2, PP3, PM1, PM2_S	PS3_Moderate	-
c.823C>T (p.Arg275Cys)	R275C	chr7:44147690:G:A	missense	Pathogenic	PP4_M, PM2_S, PS4, PP1, PP2, PP3	PS3_Supporting	Pathogenic
c.863T>G (p.Leu288Arg)	L288R	chr7:44147650:A:C	missense	Not reported	PP2, PP3, PM2_S	BS3_Supporting	-
c.941T>C (p.Leu314Pro) **	L314P	chr7:44146541:A:G	missense	Not reported	PP2, PP3, PM2_S	PS3_Moderate	-
c.1105C>G (p.Arg369Gly)	R369G	chr7:44145645:G:C	missense	Not reported	PP2	BS3_Supporting	-
c.1118G>C (p.Ser373Thr)	S373T	chr7:44145632:C:G	missense	Not reported	PP2	BS3_Supporting	-
c.1160C>T (p.Ala387Val)	A387V	chr7:44145590:G:A	missense	Pathogenic	PP4_M, PP1_St, PM2_S, PS4_M, PP2, PP3, PM5	PS3_Supporting	Pathogenic
c.1181G>T (p.Arg394Leu) **	R394L	chr7:44145569:C:A	missense	Likely pathogenic	PP4_M, PM2_S, PM5_S, PP2, PP3	PS3_Supporting	Likely pathogenic
c.1240A>G (p.Lys414Glu)	K414E	chr7:44145510:T:C	missense	Pathogenic	PP1_St, PM2_S, PS4, PM1, PP2, PP3, PP4	PS3_Supporting	Pathogenic
c.1286G>A (p.Arg429Lys)	R429K	chr7:44145248:C:T	missense	Not reported	PP2	BS3_Supporting	-
c.1348G>T (p.Ala450Ser) **	A450S	chr7:44145186:C:A	missense	Not reported	PP2, PP3, PM2_S	PS3_Moderate	-

All sequence information is based on the GenBank reference sequence NM_000162.5. Genomic positions are based on the GRCh38 build. Nucleotide numbering reflects cDNA numbering corresponding to the A of the major start codon of exon 1a, the alternate exon 1 present in the pancreatic isoform. The MDEP classifications are sourced from the ClinGen evidence repository, where available. Descriptions of criteria codes are found in the ClinGen Criteria Specific Registry. ** Variants identified in clinical diagnostic labs. _M = moderate; _St = strong; _S = supporting. ClinGen data were accessed in October 2025.

### 2.3. GCK Variant Interpretation Across In Silico Tools and Deep-Mutational Scanning Datasets

Bioinformatic predictive tools are increasingly being used to evaluate the impact of coding alleles on protein structure and function. Just like functional assays, in silico prediction tools alone cannot be used to make a classification, but are used in conjunction with other criteria in the ACMG guidelines. With improvements in artificial intelligence (AI), Google DeepMind’s AlphaFold [32], and the availability of deep mutational scanning efforts [19], we were curious to see how these tools performed in comparison to our in vitro assays. We used REVEL and AlphaMissense to classify the 23 missense variants in this study; G170_K172dup and C213del were excluded as the prediction tools are limited to point mutations (Supplementary Table S2). Using the MDEP VCEP recommendation for cut-offs, REVEL classified 15 variants as likely pathogenic (REVEL score ≥ 0.70), 8 variants as ambiguous (REVEL score > 0.15 and <0.7), and no variants as likely benign (REVEL score ≤ 0.15). We used the EBI recommendations [33] for AlphaMissense, which classified 8 variants as likely pathogenic (AlphaMissense score > 0.564), 3 variants as ambiguous (AlphaMissense score between 0.34 and 0.564), and 12 variants as likely benign (AlphaMissense score < 0.34). We also included an existing high-throughput dataset, which uses a yeast complementation assay to evaluate all possible amino acid changes in GCK [19], (Supplementary Table S2). The three tools only agreed on 6 variants (A188T, V203A, V226M, G258D, L314P, and A387V). For the 7 missense variants already evaluated by the MDEP VCEP, there was generally good concordance across modalities except for R275C, where at least two of the methods (AlphaMissense, REVEL, or high-throughput yeast assay) misclassified the variant. One variant worth highlighting is R192K. The yeast complementation assay classified it as pathogenic, whilst our in vitro data provided a criterion of BS3_supporting. There are currently insufficient data to provide a threshold for abnormal GKRP and GKA regulation, and we are limited to a binomial classification. Without a clearly defined cut-off, it is not possible to interpret the GKRP response for R192K, which does show a deviation from wild-type GCK (Figure 3).

### 2.4. GCK Allelic Spectrum for Enzyme Activity and Stability

**Figure 5:**
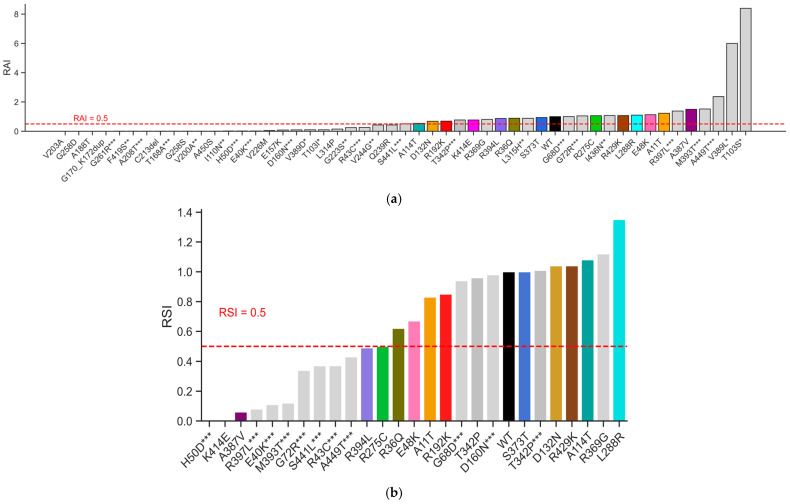
Allelic spectrum of effects for GCK variants, including previously characterized alleles. (**a**) The relative activity index (RAI) and (**b**) the relative stability index (RSI) for variants in this study are shown along with previously published variants studied with the same protocol. Wild-type (WT) is shown in black, variants from published studies are shown in gray, and those in the current study are shown in the same colors as Figure 1, Figure 2, Figure 3 and Figure 4. The current cutoff for classification for BS3/PS3 is shown for both RAI and RSI. * = variant from Beer et al. [34], ** = variant from Valentinova et al. [35], *** = variant from Raimondo et al. [11].

To illustrate the allelic spectrum for effects on both enzyme activity and stability, we combined the kinetic data from this study with our previously characterized GCK alleles [11,23,26,34,35], for a total of 50 variants for RAI and 26 for RSI (Figure 5).

## 3. Discussion

In this study, we have characterized 25 GCK variants identified through diagnostic testing (n = 5) or through exome sequencing studies in type 2 diabetes cases and controls (n = 20) using a suite of low-throughput in vitro assays that assess enzyme activity, stability, and regulation by pharmacological activators and physiological inhibitors. This data has been used to derive evidence for PS3/BS3 codes from the ACMG guidelines, which can now be combined with other evidence for variant classification. For variants that had been previously assessed by MDEP VCEP, the addition of the functional data provides further support for classification, including the ability to reclassify one variant from VUS to likely pathogenic. For the 16 variants not yet evaluated by the panel, we provide BS3_Supporting (n = 8) and PS3_Moderate (n = 8) criteria. By using the ClinGen MDEP VCEP established guidelines for variant interpretation, we standardize our functional assays for glucokinase, allowing for reproducibility across labs and groups for validating both existing and novel variants. The incorporation of gold-standard assays into the clinical guidelines strengthens the decision tree, as these kinetic assays historically remain the most reliable for quantifying enzymatic behavior and generating parameters that accurately reflect GCK’s physiological role as a metabolic glucose sensor. Our evaluation of the performance of existing in silico tools (e.g., REVEL, AlphaMissense) and the availability of existing deep-mutational scanning maps [19] demonstrates the inconsistencies and current limitations of these tools for interpreting GCK variants. Current deep-mutational scanning efforts have been performed using a yeast-complementation assay, and notably, some variants for which pathogenicity is not captured are those with complex mechanisms relating to stability and/or physiological and pharmacological regulation [19]. It has long been recognized that in silico tools have low specificity for predicting pathogenicity of coding alleles in monogenic diabetes genes and that their performance is particularly poor for gain-of-function alleles [36]. The open and closed confirmations of glucokinase, particularly around the allosteric activator site, are likely to compromise the performance of AlphaMissense [31].

Despite the use of robust, well-validated assays, there are several limitations to our study. In vitro functional assays are limited in their ability to capture cellular context. Fluctuating blood glucose levels, interactions with other components, and cellular localization can all affect a GCK variant’s ability to process glucose. There have been a small number of studies investigating molecular mechanisms for GCK variant dysfunction in rodent beta-cell lines, and these have so far aligned with assays on recombinant proteins [21,22,37,38,39]. We have used thermolability assays as a proxy for enzyme stability and titration assays to evaluate the ability of GKA and GKRP to regulate GCK variants. Although these assays have successfully identified defects for several variants in these behaviors [11,23,24], we do not have a thorough understanding of the cut-offs for these assays, nor do they assess all possible molecular mechanisms for dysfunction. For example, R275C is on the threshold for RSI (RSI ≤ 0.5), and the degree of thermolability required to cause pathogenicity has not been clearly demonstrated. The impact of compensation from the wild-type allele on the physiological manifestation of a defect in humans adds to the complexity of interpretation [13,14]. Mathematical modeling has suggested that modest instability is predicted to contribute to pathogenicity, but it needs to be taken into context with the RAI [11,14]. The current study demonstrates both the need for more data to understand the implications for pathogenicity of modest changes in thermostability and regulation by GKPR and GKA. Larger studies, which include thermostability and interactions with physiological and pharmacological regulators that capture more proven benign variants, are needed to provide more robust evidence for setting thresholds across these assays. The MDEP classification guidelines are also specifically outlined for loss-of-function variants. These cutoffs describe only lower limits, when in fact many activating or gain-of-function variants have much higher RAI or RSIs than wild-type [11,23,24,26]. Thresholds for activating *GCK* variants still need to be established and may be more complex due to a lack of compensation from the wild-type allele and challenges with defining the phenotype [14,40].

The functional pipeline outlined in this study for characterizing variants is arduous, time-consuming, and cannot keep up with the constant generation of genetic sequencing data and, in turn, the numerous VUS that are returned on diagnostic tests. It requires adaptation to allow the assessment of hundreds or thousands of *GCK* variants at once. High-throughput methodologies specifically for enzyme kinetics already exist and use microfluidics to deliver substrate to thousands of enzyme variants at a very small scale [41]. Deployment of such a technology for *GCK* would allow the assessment of every possible single-nucleotide change that results in a protein-coding variant (around 3000) and would allow a comprehensive evaluation of assay cutoffs. The growing catalog of *GCK* variants, which have been comprehensively characterized using gold-standard in vitro methods, will provide a valuable resource for calibration of these datasets.

## 4. Materials and Methods

### 4.1. Information on T2DGENES Study Candidate Variants and Clinically-Sourced Variants

Twenty-nine protein-coding candidate variants at the *GCK* locus were identified in a genome-wide association study performed on exome sequencing data of 12,940 individuals, comprising roughly equal numbers of Type 2 diabetic cases and controls across 5 ancestry groups [27]. Twenty of these variants were moved forward for functional analysis and classification in this study. These variants were synthesized, and their kinetic and stability properties were evaluated at the University of Oxford.

Clinically-sourced variants, of which there are five in this study, were identified in routine diagnostic labs performed for monogenic diabetes testing and referred by clinicians to the MDEP for further evaluation. All these variants were synthesized and characterized at Stanford University, along with variants from the T2DGENES study that required binding partner evaluation.

### 4.2. GST-GCK Construct Information

The coding sequence of human *GCK* (NM_000162.5) used is transcript variant 1, which encodes the isoform expressed in pancreatic beta cells. This sequence was cloned into a pGEX-3X vector expressing GST fusion proteins (GE Healthcare, Sunnyvale, CA, USA) and provided as a gift from Vanderbilt University (see Acknowledgments).

### 4.3. GKRP-FLAG Construct Information

The coding sequence of human *GKRP* (NM_001486) was cloned into a pFLAG_CTC vector and provided as a gift from AstraZeneca (Cambridge, UK), as previously reported [42]. The construct was sent for full plasmid sequencing to confirm the wild-type sequence of *GKRP*.

### 4.4. Site-Directed Mutagenesis

The mutations in this study were introduced to the pGEX-3X_GST-GCK construct using custom primers (Supplemental Table S3) and either the Pfu DNA Polymerase from Agilent (Santa Clara, CA, USA) (Cat: 600252) or the Q5 Mutagenesis Kit from New England Biolabs (Ipswich, MA, USA) (Cat: E0554S), following manufacturer instructions. After site-directed mutagenesis, constructs were transformed into NEB stable competent *E. coli* (Cat: C3040I) for colony picking and plasmid extraction (using Promega Pure Yield Plasmid Miniprep Kit, Promega, Madison, WI, USA, Cat: A1223). The region containing GCK was amplified, and the mutation introduced was validated by Sanger sequencing using 3GEX and 5GEX primers (Supplemental Table S4).

### 4.5. Recombinant Production of Wild-Type and Variant Human Glucokinase

GST-tagged human GCK protein was generated from the GST-GCK construct in three separate labs (Oxford, Stanford-Fordyce, and Stanford-Gloyn). The following protocol was generally used by all labs unless otherwise noted. BL21 E. coli was transformed with the validated pGEX-3X_GST-GCK construct, and cultures were grown to either 500 mL or 2 L in Luria broth with ampicillin or carbenicillin (100 µg/mL working concentration). In Oxford, beta-mercaptoethanol was added to the cells during transformation. Once the optical density of the cultures reached 0.6–0.8 at 600 nm, protein production was induced by IPTG (final concentration 0.3 mM, Cat: sc-202185B). In Oxford, the final concentration of IPTG used was 0.2 mM. After overnight induction at room temperature, the cultures were spun down at 4000× *g* for 10–20 min, and pellets were resuspended in 10–15 mL lysis buffer (PBS pH 7.4, 5 mM DTT, 2× EDTA-free protease inhibitor—Roche #11697498001, Basel, Switzerland) and lysed using either a sonicator (Stanford-Gloyn, Oxford) or a high-pressure homogenizer (Stanford-Fordyce). At Stanford, the sonicator was used at 30% amplitude for 4 blasts at 30 s at a time, for a total of 2 min. In Oxford, the sonicator was set at 0.17 intensity for 45 s blasts, for a total of 5 min. 100 µL of Triton X-100 was added to the lysate and spun down at 21,100× *g* at 4 °C for 30 min (or 5000× *g* for 8 min at Oxford). Cell debris was discarded, and the supernatant was filtered through a 0.2 μm filter (no filtration performed at Oxford), then incubated for 2 min at room temperature with 1 mL (1 CV) glutathione agarose resin (Fisher Scientific #501532778, Hampton, NH, USA). In Oxford, each lysate was incubated with 6 mL of 50% resin. The supernatant and resin were loaded onto a gravity-flow column. The column was washed with 15–30 CV of lysis buffer (PBS + DTT + PIC) and eluted with 5–10 individually collected CVs of elution buffer (50 mM Tris-HCl, pH 8.0, 200 mM KCl, 5 mM DTT, and 10 mM glutathione). At Stanford-Gloyn, the first CV of the elution buffer was incubated for 1 h before elution. Purified GST-GCK was verified by SDS-PAGE (bands at ~76 kDa), and concentration was measured by Bradford assay. Eluted protein was mixed with a storage buffer (50 mM Tris-HCl, pH 7.5, 200 mM KCl, 60% glycerol, 100 mM glucose, and 5 mM DTT) at a 2:3 ratio, aliquoted, and saved at −80 °C. At Oxford, the ratio of eluted protein to storage buffer was 1:1. Protein purity was established by SDS PAGE (Supplementary Figure S2).

### 4.6. Recombinant Production of Wild-Type Human Glucokinase Regulatory Protein

FLAG-tagged human GKRP was generated from the GKRP_FLAG construct at Stanford University. The protocol for generating recombinant GCK at Stanford–Gloyn was followed except for the following changes. Six liters of transformed BL21 culture were generated. The final concentration of IPTG was 0.6 mM. Sodium chloride was added to the filtered lysate at a final concentration of 0.2 M, and the pH was adjusted to 7–8 if needed. Anti-FLAG M2 affinity resin (Sigma #A2220, St. Louis, MO, USA) was then incubated with the lysate for 1 h at 4 °C with end-over-end gentle shaking. The elution buffer was prepared by resuspending 3× FLAG peptide (Thermofisher #A36805, Waltham, MA, USA) in PBS at a concentration of 1.5 mg/mL. The storage buffer was identical to the GCK storage buffer except that no glucose was included, as glucose is inhibitory to GKRP. On SDS-PAGE, bands were checked at ~70 kDa for validation of purified GKRP-FLAG, and concentration was measured by Bradford assay.

### 4.7. Glucose- and ATP-Dependent Assays

GCK wild-type and variant activity was measured using a coupled enzymatic reaction that used glucose-6-phosphate dehydrogenase to dehydrogenate glucose-6-phosphate to a lactone and generate NADPH in the process, which can be measured at 340 nm as previously described [11,20,23]. Kinetic assays were performed on a multi-mode microplate reader (SpectraMax iD3, Molecular Devices, San Jose, CA, USA) at 30 °C in a final volume of 200 µL, in Assay Buffer (100 mM HEPES pH 7.4, 6 mM MgCl_2_, 150 mM KCl, 0.1% BSA (ThermoFisher #AM2616), 1 mM NADP+ (Santa Cruz Biotech #sc-215561, Santa Cruz, CA, USA), 7.81 nM G6PDH (Sigma #G6378, ThermoScientific #J611814I), 2 mM DTT), and 1 nM GCK. Titrations were carried out at 0–100 mM glucose and 5 mM ATP for glucose-dependent assays, and at 0–5 mM ATP and 100 mM glucose for ATP-dependent assays. At Oxford, variants were assayed at their S_0.5_ glucose concentrations for ATP titrations as opposed to saturating concentrations. Variants that had very high S_0.5_s and/or ATP *k*_M_s were assayed again with larger glucose or ATP titration ranges to ensure the enzyme was saturated at the highest concentration. The following enzymes deviated from the normal ranges: E157K (0–200 mM glucose), A188T (0–1000 mM glucose, 0–50 mM ATP), V226M (0–1000 mM, 0–25 mM ATP), and G258S (0–1000 mM glucose, 0–25 mM). Saturation curves, where available, are provided in Supplementary Figure S3.

Purified GCK was first incubated in assay buffer and glucose for 30 min at 30 °C, then ATP was added, and the absorbance of the reaction progression was measured at 340 nm to record NADPH generation over time, as a proxy for GCK activity. Enzymatic rates at each substrate concentration were fit to the Hill equation (for glucose titrations) and Michaelis–Menten (for ATP titrations) to generate S_0.5_/ATP *k*_m_ values, Vmax values (maximum enzyme speed), and Hill numbers (for glucose titrations).

### 4.8. Thermolability Assays

Purified wild-type and variant GCK were diluted to 20 nM with Incubation Buffer (100 mM HEPES pH 7.4, 6 mM MgCl_2_, 150 mM KCl, 0.1% BSA, 2 mM DTT) and saturating concentrations of glucose. This mix was then incubated at 12 different temperatures between 40 and 63 °C (Oxford) or 35–52 °C (Stanford) for 30 min. The discrepancy in temperature range was due to variation in lab settings—variant controls were run with every assay to anchor the assay in place and ensure previously established data did not change between labs. Kinetic assays were then performed by diluting the incubated GCK to 1–5 nM in Assay Buffer (described above), with saturating ATP concentrations and S_0.5_ glucose concentrations in a final volume of 200 µL. NADPH generation was measured over time, and enzymatic rates at each temperature were fit to a 4-parameter logistic regression to generate TA50 values.

### 4.9. Calculation of Relative Activity Index

The following equation was used to calculate the activity index for each variant [14,20,43].

(1) $$\frac{k_{cat}}{S_{0.5}^{\;Hill}}\times \frac{\left[ATP\right]}{\left[ATP\right]+ATP_{Km}}\times \frac{2\times \left(5^{Hill}\right)}{5^{Hill}+S_{0.5}^{\;Hill}},$$

This equation accounts for and assumes that the intracellular concentration of ATP is 2.5 mM. It also accounts for the expression of GCK at a basal blood glucose of 5 mM. The activity index is then normalized to that of the wild-type.

### 4.10. Calculation of Relative Stability Index

The following equation was used to calculate the stability index for each variant relative to wild-type [11].

(2) $$\frac{TA50_{mutant}-TA50_{min}}{TA50_{wild-type}-TA50_{min}},$$

TA50_min_ refers to the minimum TA50 observed in this study.

### 4.11. Glucokinase Regulatory Protein Inhibition Assays

Purified wild-type and variant GCK (1 nM) were incubated at 30 °C for 30 min in Assay Buffer at 3 mM glucose and 0–60 nM GKRP. After incubation, ATP was added to the mixture at a final concentration of 5 mM and a final reaction volume of 200 µL. NADPH generation was measured over time, and enzymatic rates were plotted against GKRP concentration.

### 4.12. Small Molecule Activation Assays

Purified wild-type and variant GCK (1 nM) were incubated at 30 °C for 30 min in Assay Buffer and 0–100 mM glucose. GK-activator (RO-28-1675 in DMSO, Sigma #5096650001) was titrated at a final concentration of 0–60 µM, and GCK plus activator were left to incubate in assay buffer and glucose for 5 min at 30 °C. ATP was then added to each well at a final concentration of 5 mM. NADPH generation was measured over time, and enzymatic rates at 3.125 mM and 6.25 mM glucose were plotted against each GK-activator concentration. These curves were fit to a 4-parameter logistic regression to generate EC50 values at 3.125 and 6.25 mM glucose concentrations.

### 4.13. Glucose-Stimulated Insulin Secretion Threshold Calculation

The model for predicting the threshold for glucose-stimulated insulin secretion (GSIS) from the pancreatic beta cell for a given heterozygous variant is previously described [14]. It makes a number of assumptions: (1) GCK in beta cells controls GSIS, (2) for wild-type GCK, the physiological threshold for GSIS is 5 mM, and the beta cell glucose phosphorylation rate (BGPR) is 28%, (3) glucose and ATP dependency of the BGPR is determined by the Hill equation and Michaelis–Menten kinetics, respectively, and (4) blood glucose concentration alters the expression of the wild-type and mutant allele. Kinetic parameters obtained from glucose- and ATP-dependent assays, as described, were input into the equation to estimate G (GSIS threshold). For variants that are thermolabile, the enzyme stability coefficient (S) was adjusted from 1 (for stable variants) to reflect the effect of enzyme instability on GSIS thresholds.

## Figures and Tables

**Table 1:** Kinetic characterization of 25 GCK variant proteins using gold-standard kinetic in vitro assays.

Variant	Yield (mg)	Glucose S_0.5_ (mM)	ATP *k_M_* (mM)	Hill n_H_	*k_cat_*^[A]^ (s^−1^)	*k_cat_*^[B]^ (s^−1^)	*k_cat_*^[A]^/S_0.5_ (s·mM^−1^)	Predicted GSIS Threshold (mM)	RAI	TA50 (°C)	RSI	Ro-28-1675 EC_50_ (μM) ^†^	GKRP Y/N ^†^	Functional Evidence (Figure 1f)
Variants assessed in laboratory at Oxford University														
WT	4.42	7.32 ± 0.03	0.55 ± 0.01	1.70 ± 0.01	63.96 ± 1.08	62.44 ± 1.93	8.73	5.00	1.00	54.92	1.00	-	-	-
A11T	20.23	7.04 ± 0.14	0.57 ± 0.01	1.61 ± 0.03	57.42 ± 1.02	52.25 ± 0.83	8.16	4.93	1.23	54.43	0.83	1.79 ± 0.11	Y	BS3_Supporting
R36Q	18.98	7.90 ± 0.14	0.58 ± 0.01	1.62 ± 0.02	59.69 ± 0.99	53.62 ± 0.99	7.56	5.21	0.89	53.82	0.62	1.97 ± 0.24	Y	BS3_Supporting
E48K	13.85	7.03 ± 0.07	0.60 ± 0.01	1.64 ± 0.02	56.11 ± 0.71	53.79 ± 0.66	7.98	4.97	1.13	53.96	0.67	1.51 ± 0.08	Y	BS3_Supporting
A114T	11.15	7.53 ± 0.09	0.78 ± 0.02	1.71 ± 0.02	41.16 ± 0.63	42.85 ± 0.29	5.47	5.58	0.54	55.16	1.08	2.01 ± 0.34	Y	BS3_Supporting
D132N	9.66	8.01 ± 0.10	0.52 ± 0.01	1.63 ± 0.02	46.38 ± 1.18	39.73 ± 0.71	5.79	5.45	0.68	55.05	1.04	1.98 ± 0.36	Y	BS3_Supporting
E157K	10.74	18.97 ± 0.23	0.46 ± 0.01	1.61 ± 0.02	58.63 ± 0.79	51.82 ± 0.87	3.09	6.67	0.08 *	ND	ND	ND	ND	PS3_Moderate
A188T	10.83	106.64 ± 4.07	2.85 ± 0.06	1.18 ± 0.02	23.22 ± 0.20	15.67 ± 0.17	0.22	7.80	0.00 *	ND	ND	ND	ND	PS3_Moderate
R192K	6.66	7.81 ± 0.09	0.37 ± 0.01	1.77 ± 0.02	58.08 ± 0.43	49.47 ± 0.49	7.44	5.19	0.69	54.48	0.85	3.61 ± 0.13	Y	BS3_Supporting
V203A	-	N/A	N/A	N/A	N/A	N/A	N/A	N/A	0.00 *	ND	ND	ND	ND	PS3_Moderate
V226M	3.23	41.63 ± 0.91	3.02 ± 0.05	1.11 ± 0.01	51.3 ± 0.75	60.58 ± 0.89	1.23	6.97	0.05 *	ND	ND	ND	ND	PS3_Moderate
Q239R	8.48	7.86 ± 0.15	0.56 ± 0.01	1.75 ± 0.02	37.47 ± 0.32	37.45 ± 0.41	4.77	5.93	0.43 *	ND	ND	ND	ND	PS3_Moderate
G258S	11.87	327.41 ± 9.21	4.28 ± 0.11	0.88 ± 0.00	68.97 ± 0.62	38.72 ± 0.43	0.21	7.08	0.01 *	ND	ND	ND	ND	PS3_Moderate
G258D	-	N/A	N/A	N/A	N/A	N/A	N/A	N/A	0.00 *	ND	ND	ND	ND	PS3_Moderate
R275C	10.30	8.05 ± 0.09	0.55 ± 0.00	1.62 ± 0.01	71.72 ± 0.87	62.47 ± 0.94	8.91	5.00	1.06	53.46	0.50 *	2.85 ± 0.42	Y	PS3_Supporting
L288R	22.50	6.48 ± 0.05	0.50 ± 0.00	1.70 ± 0.01	48.42 ± 0.67	45.16 ± 0.34	7.48	4.94	1.10	55.93	1.35	1.56 ± 0.18	Y	BS3_Supporting
R369G	3.71	7.09 ± 0.06	0.67 ± 0.02	1.64 ± 0.01	42.54 ± 0.54	41.52 ± 0.48	6.00	6.16	0.81	55.27	1.12	2.24 ± 0.57	Y	BS3_Supporting
S373T	16.12	7.88 ± 0.11	0.66 ± 0.03	1.63 ± 0.02	63.78 ± 1.24	52.89 ± 0.95	8.09	5.13	0.94	54.91	1.00	2.87 ±0.16	Y	BS3_Supporting
A387V	0.77	5.31 ± 0.06	0.58 ± 0.00	1.59 ± 0.02	32.71 ± 1.27	28.67 ± 1.10	6.16	>7.00	1.50	52.19	0.06 *	ND	ND	PS3_Supporting
K414E	3.30	6.56 ± 0.15	1.24 ± 0.03	1.63 ± 0.01	43.32 ± 0.72	45.66 ± 0.67	6.60	>7.00	0.77	52.02	0.00	ND	ND	PS3_Supporting
R429K	16.60	7.00 ± 0.10	0.60 ± 0.02	1.65 ± 0.03	54.1 ± 0.99	44.09 ± 0.47	7.73	5.01	1.07	55.03	1.04	2.81 ± 0.11	Y	BS3_Supporting
Variants assessed in the laboratory at Stanford University														
WT	3.36	7.18 ± 0.10	0.46 ± 0.01	1.71 ± 0.01	54.24 ± 1.27	58.60 ± 1.68	7.55	5.00	1.00	47.10	1.00	2.30 ± 0.44	-	-
G170_K172dup	3.50	N/A	N/A	N/A	N/A	N/A	N/A	N/A	0.00 *	ND	ND	ND	ND	PS3_Moderate
C213del	1.34	34.85 ± 3.18	2.23 ± 0.17	1.36 ± 0.04	11.62 ± 0.83	17.18 ± 0.98	0.33	6.66	0.01 *	ND	ND	ND	ND	PS3_Moderate
L314P	0.35	9.03 ± 0.27	0.49 ± 0.01	1.77 ± 0.04	18.63 ± 2.58	21.26 ± 3.12	2.06	6.25	0.15 *	ND	ND	ND	ND	PS3_Moderate
R394L	3.39	7.39 ± 0.29	0.48 ± 0.01	1.67 ± 0.01	47.09 ± 1.36	61.02 ± 5.19	6.38	>7.00	0.87	45.48	0.49 *	ND	ND	PS3_Supporting
A450S	3.62	23.23 ± 0.97	0.56 ± 0.02	1.59 ± 0.05	14.21 ± 1.04	16.88 ± 1.04	0.61	6.25	0.01 *	ND	ND	ND	ND	PS3_Moderate

All stability measurements were generated in the Oxford laboratory except for R394L, which was generated at Stanford. ^†^ Activator and ^†^ GKRP binding assays were all performed in the Stanford lab. Glucose S_0.5_, Hill number, and *k*_cat_^[A]^ values were determined in the presence of 0–100 mM glucose unless otherwise indicated in the materials and methods. The ATP *k*_M_ was determined in the presence of 0–5 mM ATP unless otherwise indicated in the Materials and Methods section. *k*_cat_^[A]^ values were determined from glucose titration assays, and *k*_cat_^[B]^ values were determined from ATP titrations. Data shown are mean ± SEM (n = 3 biological replicates, 3 technical replicates) for all variants evaluated at Stanford and n = 1 biological and ≥3 technical replicates for all variants evaluated at Oxford. EC50 values were measured at 3.125 mM glucose. N/A, not available—for G170_K172dup, V203A, and G258S, the affinity for glucose or ATP was so low that S_0.5_, ATP *k*_M_, Hill number, and *k_cat_* could not be measured, even at high substrate concentrations. ND, not determined—a classification could be determined for that variant without needing that column’s data. Predicted threshold or GSIS was calculated using the published mathematical model^14^. For thermolabile proteins A387V, R394L, and K414E, the stability coefficient was set to 0.1 to model the impact of GSIS. GKRP regulation is either normal (comparable to wildtype, Yes (Y)) or No (N). Functional evidence was determined by deploying the MDEP VCEP decision tree for GCK functional assays depicted in Figure 1f. Values which are supportive of a functional defect are shown in bold *.

## Data Availability

The original contributions presented in this study are included in the article/Supplementary Material. Further inquiries can be directed to the corresponding author.

## Supplementary Materials

The following supporting information can be downloaded at: https://www.mdpi.com/article/10.3390/ijms27010156/s1.

## Abbreviations

The following abbreviations are used in this manuscript:

GCK	Glucokinase
GKRP	Glucokinase Regulatory Protein
RAI	Relative Activity Index
RSI	Relative Stability Index
MDEP	Monogenic Diabetes Expert Panel
VCEP	Variant Curation Expert Panel
ClinGen	Clinical Genome Resource
ClinVar	Clinical Variation
GSIS	Glucose-Stimulated Insulin Secretion
LoF	Loss of Function
GoF	Gain of Function
MODY	Maturity-Onset Diabetes of the Young
PNDM	Permanent Neonatal Diabetes Mellitus
TNDM	Transient Neonatal Diabetes Mellitus
ACMG/AMP	American College of Medical Genetics and Genomics/Association for Molecular Pathology
VUS	Variant of Unknown Significance
T2DGENES	Type 2 Diabetes Genetic Exploration by Next-Generation Sequencing in Multi-Ethnic Samples
PHHI	Persistent Hyperinsulinemic Hypoglycemia of Infancy
WT	Wild-type
REVEL	Rare Exome Variant Ensemble Learner
HVGS	Human Genome Variation Society
GST	Glutathione-s-transferase
IPTG	Isopropyl-β-D-thiogalactopyranoside
CV	Column Volume
ATP	Adenine Triphosphate
NADPH	Nicotinamide Adenine Dinucleotide Phosphate
G6PDH	Glucose-6-phosphate Dehydrogenase
BGPR	Beta-cell Glucose Phosphorylation Rate
