# Supplementary material for: Functional Characterization of Glucokinase Variants to Aid Clinical Interpretation of Monogenic Diabetes
Source: Int J Mol Sci. 2025 Dec 23;27(1):156. doi: 10.3390/ijms27010156 (PMC12785307; doi:10.3390/ijms27010156)
Supplement: Supplementary file 1 [file ijms-27-00156-s001.zip › ijms-4022464-supplementary.pdf]

International Journal of *Molecular Sciences*

MDPI

Supplementary Online Information

Supplementary Table S1: Information from ClinVar where available for the 25 variants included in the study.

Variant (HGVS)	1-Letter Code	Genomic Position (GRCh38)	Molecular Consequence	ClinVar ID	Aggregate classification	ClinVar evidence
c.31G>A (p.Ala11Thr)	A11T	chr7:44188923:C:T	missense	VCV000129141.22	Benign	Multiple reports: 9 benign
c.107G>A (p.Arg36Gln)	R36Q	chr7:44153402:C:T	missense	VCV000972809.9	VUS	Multiple reports: 4 VUS
c.142G>A (p.Glu48Lys)	E48K	chr7:44153367:C:T	missense	VCV001299600.5	Likely benign	Multiple reports: 1 likely benign; 3 VUS
c.340G>A (p.Ala114Thr)	A114T	chr7:44152294:C:T	missense	-		
c.394G>A (p.Asp132Asn)	D132N	chr7:44151045:C:T	missense	VCV000972807.5	VUS	Multiple reports: 3 VUS
c.469G>A (p.Glu157Lys)	E157K	chr7:44150970:C:T	missense	VCV000447402.29	Conflicting	Multiple reports: 5 pathogenic; 3 likely pathogenic; 2 VUS
c.509_517dup (p.Gly170_Lys172dup)**	G170_K172dup	N/A	insertion	-		
c.562G>A(p.Ala188Thr)	A188T	chr7:44149986:C:T	missense	VCV000804849.27	Pathogenic	Multiple reports: 10 pathogenic
c.575G>A(p.Arg192Lys)	R192K	chr7:44149973:C:T	missense	-		
c.608T>C (p.Val203Ala)	V203A	chr7:44149831:A:G	missense	VCV000585923.19	Pathogenic	Multiple reports: 8 pathogenic; 1 VUS
c.638_640delGCT** (p.Cys213del)	C213del	chr7:44149799:AGC:-	deletion	VCV000804853.7	VUS	Multiple reports: 4 VUS
c.676G>A (p.Val226Met)	V226M	chr7:44149763:C:T	missense	VCV000036243.38	Pathogenic	Multiple reports: 13 pathogenic; 1 likely pathogenic
c.716A>G (p.Gln239Arg)	Q239R	chr7:44147797:T:C	missense	VCV000972804.3	VUS	Multiple reports: 2 VUS
c.772G>A (p.Gly258Ser)	G258S	chr7:44147741:C:T	missense	VCV000918070.8	Conflicting	Multiple reports: 1 pathogenic; 1 likely pathogenic; 1 VUS
c.773G>A (p.Gly258Asp)	G258D	chr7:44147740:C:T	missense	VCV000972775.3	Conflicting	Multiple reports: 1 likely pathogenic; 1 VUS
c.823C>T (p.Arg275Cys)	R275C	chr7:44147690:G:A	missense	VCV000585927.5	Pathogenic	Multiple reports: 3 pathogenic, 1 VUS
c.863T>G (p.Leu288Arg)	L288R	chr7:44147650:A:C	missense	-		
c.941T>C (p.Leu314Pro)**	L314P	chr7:44146541:A:G	missense	VCV000435305.9	Conflicting	Multiple reports: 1 pathogenic, 1 likely risk, 1 VUS
c.1105C>G (p.Arg369Gly)	R369G	chr7:44145645:G:C	missense	VCV003384948.1	VUS	Single report: VUS
c.1118G>C (p.Ser373Thr)	S373T	chr7:44145632:C:G	missense	-		
c.1160C>T (p.Ala387Val)	A387V	chr7:44145590:G:A	missense	VCV000036182.9	Pathogenic	Multiple reports: 1 pathogenic, 2 likely pathogenic, 2 VUS
c.1181G>T (p.Arg394Leu)**	R394L	chr7:44145569:C:A	missense	VCV001522625.8	Likely pathogenic	Multiple reports: 1 likely pathogenic, 1 VUS
c.1240A>G (p.Lys414Glu)	K414E	chr7:44145510:T:C	missense	VCV000036188.14	Pathogenic	Multiple reports: 4 pathogenic, 4 likely pathogenic
c.1286G>A (p.Arg429Lys)	R429K	chr7:44145248:C:T	missense	-		
c.1348G>T (p.Ala450Ser)**	A450S	chr7:44145186:C:A	missense	VCV001770532.6	Pathogenic/likely pathogenic	Multiple reports: 1 pathogenic, 2 likely pathogenic

*All sequence information is based on the GenBank reference sequence NM_000162.5. Genomic positions are based on the GRCh38 build. Nucleotide numbering reflects cDNA numbering corresponding to the A of the major start codon of exon 1a, the alternate exon 1 present in the pancreatic isoform. **Variants identified in clinical diagnostic labs. ClinVar evidence includes all studies listed in ClinVar as sourced in November 2025. Aggregate classification per ClinVar does not necessarily consider all listed studies.

Supplementary Table S2: Classification of variants from a published deep mutational scanning assay and 2 in silico prediction tools.

Variant	Gersing Activity Scores	AlphaMissense	REVEL	PS3/BS3 Interpretation using Functional Evidence (fig 1f)
A11T	0.658 ± 0.304	Likely benign (0.0933)	Ambiguous (0.313)	BS3_Supporting
R36Q	0.832 ± 0.237	Likely benign (0.1442)	Likely pathogenic (0.833)	BS3_Supporting
E48K	NA	Likely benign (0.1185)	Ambiguous (0.696)	BS3_Supporting
A114T	1.129 ± 0.425	Likely benign (0.1938)	Likely pathogenic (0.730)	BS3_Supporting
D132N	0.718 ± 0.603	Likely benign (0.1302)	Ambiguous (0.465)	BS3_Supporting
E157K	-0.134 ± 0.166	Likely benign (0.2154)	Ambiguous (0.688)	PS3_Moderate
G170_K172dup	NA	NA	NA	PS3_Moderate
A188T	0.394 ± 0.250	Likely pathogenic (0.8523)	Likely pathogenic (0.933)	PS3_Moderate
R192K	0.368 ± 0.325	Ambiguous (0.3725)	Likely pathogenic (0.720)	BS3_Supporting
V203A	-0.211 ± 0.543	Likely pathogenic (0.8324)	Likely pathogenic (0.946)	PS3_Moderate
C213del	NA	NA	NA	PS3_Moderate
V226M	0.549 ± 0.205	Likely pathogenic (0.7465)	Likely pathogenic (0.789)	PS3_Moderate
Q239R	1.184 ± 0.441	Likely benign (0.0625)	Ambiguous (0.495)	PS3_Moderate
G258S	1.085 ± 1.551	Likely pathogenic (0.9559)	Likely pathogenic (0.994)	PS3_Moderate
G258D	-0.575 ± 1.194	Likely pathogenic (0.9907)	Likely pathogenic (0.990)	PS3_Moderate
R275C	0.563 ± 0.158	Likely benign (0.1845)	Likely pathogenic (0.901)	PS3_Supporting
L288R	1.188 ± 0.690	Likely benign (0.1166)	Likely pathogenic (0.753)	BS3_Supporting
L314P	0.351 ± 0.358	Likely pathogenic (0.9808)	Likely pathogenic (0.919)	PS3_Moderate
R369G	0.652 ± 0.211	Likely benign (0.2403)	Ambiguous (0.679)	BS3_Supporting
S373T	0.987 ± 0.223	Likely benign (0.097)	Ambiguous (0.504)	BS3_Supporting
A387V	0.087 ± 0.13	Likely pathogenic (0.6817)	Likely pathogenic (0.871)	PS3_Supporting
R394L	0.588 ± 0.089	Ambiguous (0.4223)	Likely pathogenic (0.801)	PS3_Supporting
K414E	0.886 ± 0.165	Likely pathogenic (0.9492)	Likely pathogenic (0.928)	PS3_Supporting
R429K	1.015 ± 0.253	Likely benign (0.0971)	Ambiguous (0.285)	BS3_Supporting
A450S	-0.242 ± 0.375	Ambiguous (0.5335)	Likely pathogenic (0.848)	PS3_Moderate

*Gersing activity scores are taken from Gersing et al 2023[15], which describes a high throughput yeast complementation assay. Activity scores represent the variant's impact on yeast growth, with the threshold for GCK-MODY set at < 0.66 and HH set at > 1.18. The in-silico tools were run as plugins in Ensembl VEP (v112), with annotation files downloaded from their respective websites and default parameters applied as recommended by the plugin documentation. Cutoffs for predictive tools are as follows: REVEL – Likely pathogenic ≥ 0.7 ≥ Ambiguous ≥ 0.15 Likely benign, AlphaMissense - Likely pathogenic ≥ 0.564 > Ambiguous > 0.34 ≥ Likely benign. Consistent classifications across the 3 are shown in green, conflicting in red.

Supplementary Table S3: Primers used for site-directed mutagenesis.

Variant	Forward Primer (5′ to 3′)	Reverse Primer (5′ to 3′)
A11T	AGCCAGGATGGAGGCCACCAAGAAGGAGAAGGTAGAGC	GCTCTACCTTCTCCTTCTTGGTGGCCTCCATCCTGGCT
R36Q	CTGAAGAAGGTGATGAGACAGATGCAGAAGGAGATGG	CCATCTCCTTCTGCATCTGTCTCATCACCTTCTTCAG
E48K	ACCGCGGCCTGAGGCTGAAGACCCATGAAGAGGCC	GGCCTCTTCATGGGTCTTCAGCCTCAGGCCGCGGT
A114T	TACTCCATCCCCGAGGACACCATGACCGGCACTGC	GCAGTGCCGGTCATGGTGTCCTCGGGGATGGAGTA
D132N	CATCTCTGAGTGCATCTCCAACTTCCTGGACAAGC	GCTTGTCCAGGAAGTTGGAGATGCACTCAGAGATG
E157K	CCTTTCCTGTGAGGCACAAAGACATCGATAAGGGC	GCCCTTATCGATGTCTTTGTGCCTCACAGGAAAGG
G170_K172dup	CAAGGCCTCAGGAGCAGAAGGGAACAATGTCGTGGGGCT	AAGCCCTTGAAGCCCTTGGTCCAGTTGAGAAGGATGCCC
A188T	GTGGGGCTTCTGCGAGACACTATCAAACGGAGAGGGG	CCCCTCTCCGTTTGATAGTGTCTCGCAGAAGCCCCAC
R192K	GACGCTATCAAACGGAAAGGGGACTTTGAAATGG	CCATTTCAAAGTCCCCTTTCCGTTTGATAGCGTC
V203A	GGATGTGGTGGCAATGGCGAATGACACGGTGGC	GCCACCGTGTCATTCGCCATTGCCACCACATCC
C213del	ACTACGAAGACCATCAGTGCGAGGT	AGGAGATCATCGTGGCCACCGTGTC
V226M	GCGAGGTCGGCATGATCATGGGCACGGGCTGCAATGCC	GGCATTGCAGCCCGTGCCCATGATCATGCCGACCTCGC
Q239R	GCTACATGGAGGAGATGCGGAATGTGGAGCTGGTGGAG	CTCCACCAGCTCCACATTCCGCATCTCCTCCATGTAGC
G258S	GCGTCAATACCGAGTGGAGCGCCTTCGGGGACTCCGGC	GCCGGAGTCCCCGAAGGCGCTCCACTCGGTATTGACGC
G258D	CGTCAATACCGAGTGGGACGCCTTCGGGGACTCCGG	CCGGAGTCCCCGAAGGCGTCCCACTCGGTATTGACG
R275C	GTTCCTGCTGGAGTATGACTGCCTGGTGGACGAGAGC	GCTCTCGTCCACCAGGCAGTCATACTCCAGCAGGAAC
L288R	GCAAACCCCGGTCAGCACGGTATGAGAAGCTCATAGG	CCTATGAGCTTCTCATACCGTGCTGACCGGGGTTTGC
L314P	AGGCTCGTGGACGAAAACCCGCTCTTCCACGGGGAG	CTCCCCGTGGAAGAGCGGGTTTTCGTCCACGAGCCT
R369G	CGACTGCGACATCGTGCGCGGCGCCTGCGAGAGCGTGTC	GACACGCTCTCGCAGGCGCCGCGCACGATGTCGCAGTCG
S373T	CGCCGGCGCCTGCGAGACCGTGTCTACGCGCGCTGC	GCAGCGCGCGTAGACACGGTCTCGCAGGCGCCGGCG
A387V	GTGCTCGGCGGGGCTGGTGGGCGTCATCAACCGCATGC	GCATGCGGTTGATGACGCCCACCAGCCCCGCCGAGCAC
R394L	GGCGTCATCAACCGCATGCTCGAGAGCCGCAGCGAG	CTCGCTGCGGCTCTCGAGCATGCGGTTGAT GACGCC
K414E	CGTGGATGGCTCCGTGTACGAGCTGCACCCCAGCTTCAAG	CTTGAAGCTGGGGTGCAGCTCGTACACGGAGCCATCCACG
R429K	CCATGCCAGCGTGCGCAAGCTGACGCCCAGCTGCGAG	CTCGCAGCTGGGCGTCAGCTTGCGCACGCTGGCATGG
A450S	AGTGGCCGGGGCGCGTCCCTGGTCTCGGCGGTGGAG	CACCGCCGAGACCAGGGACGCGCCCCGGCCACT

*Forward and reverse primers for site-directed mutagenesis.

Supplementary Table S4: Primers used for Sanger sequencing validation.

Primer	Sequence (5′ to 3′)
5GEX	GGGCTGGCAAGCCACGTTTGGTG
3GEX	CCGGGAGCTGCATGTGTCAGAGG

*Forward and reverse primers for PCR amplification of the GCK fragment within the pGEX-3X vector for sanger sequencing validation.

Supplementary Figure S1

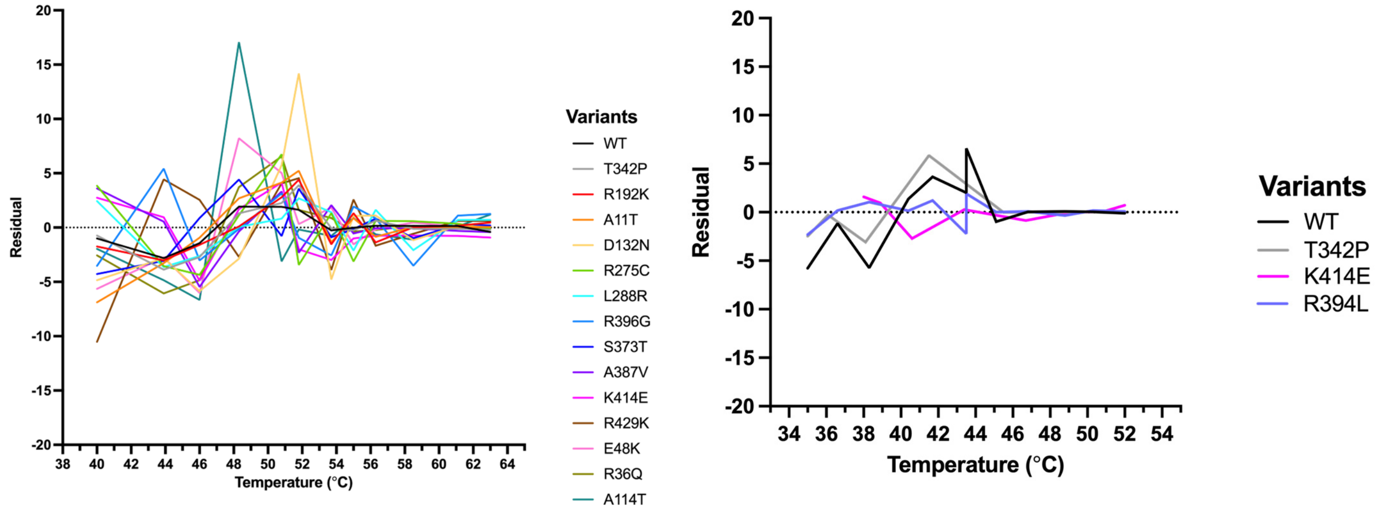

(a) (b)

Figure S1. Residual plots for the four-parameter logistic regression thermostability curves shown in Figure 2 in the main text. Residuals (observed – predicted) values are plotted against temperature for each variant. The dotted line at 0 indicates a perfect logistic regression fit. [a] Residual plots for variants assessed at Oxford. [b] Residual plots for variants assessed in Stanford.

Supplementary Figure S2

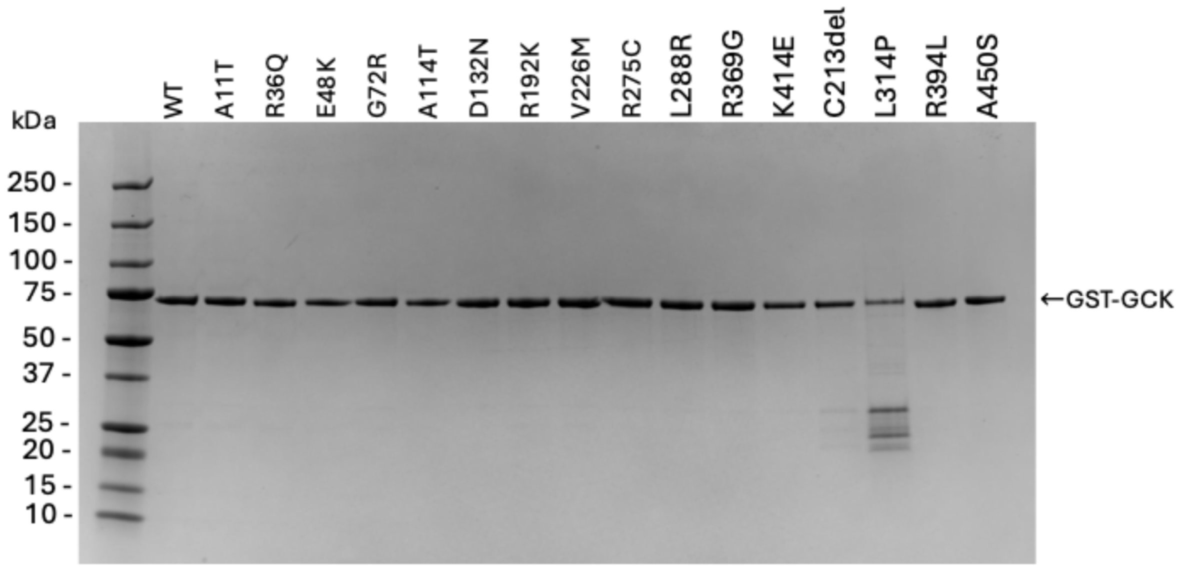

Figure S2. SDS-page gel results for purified recombinant enzyme for 17 variants including wildtype. GCK recombinant enzyme after purification and combined with storage buffer was run on an SDS-page gel (4-20%) at 200 V. Bands at 75 kD are indicative of GST-tagged glucokinase which has a molecular weight of about 75 kD.

Supplementary Figure S3

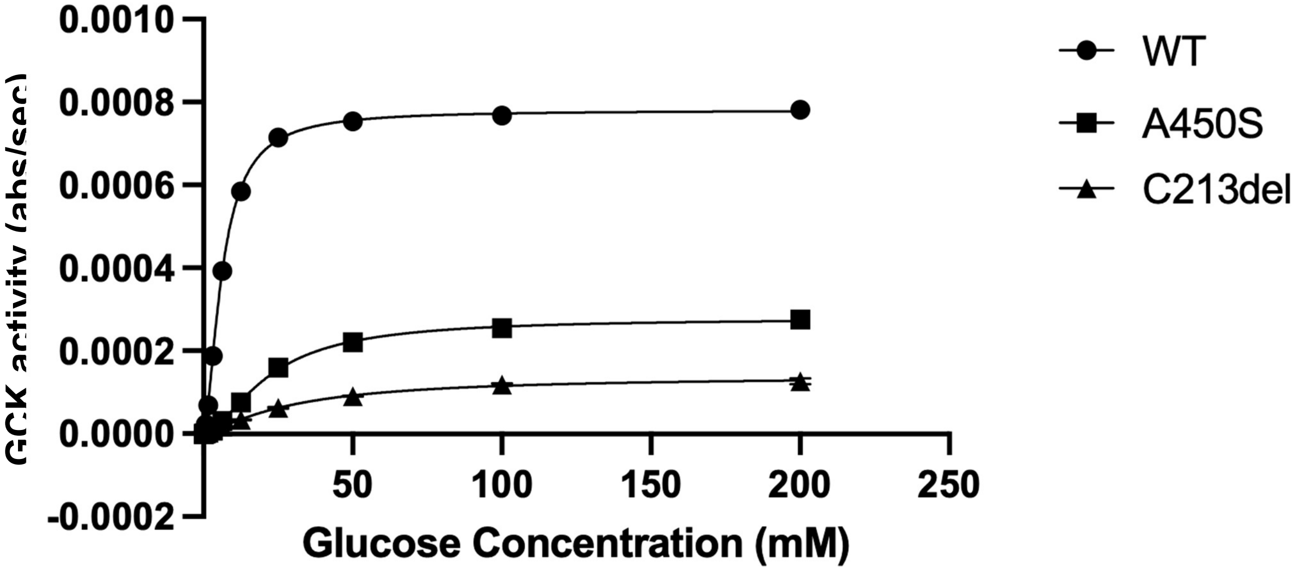

Figure S3. Saturation curves for two glucokinase variants. Increased glucose concentrations at saturating ATP levels (>5 mM) are needed to reach saturation for certain inactivating variants, two of which are shown here. While 100 mM glucose is sufficient to saturate wildtype, at least 200 mM glucose is necessary for both A450S and C213del to reach saturation (n = 1).
